# Current use of push‐dose epinephrine: Survey and interview results from academic clinicians in emergency medicine

**DOI:** 10.1111/acem.70014

**Published:** 2025-03-18

**Authors:** Andrew K. Posen, Shaveta Khosla, Terry Vanden Hoek, Renee M. Petzel Gimbar

**Affiliations:** ^1^ Department of Emergency Medicine University of Illinois Hospital and Health Sciences System (UI Health) Chicago Illinois USA; ^2^ Department of Pharmacy Practice University of Illinois Chicago, College of Pharmacy Chicago Illinois USA; ^3^ Department of Emergency Medicine University of Illinois Chicago, College of Medicine Chicago Illinois USA

Emergency department (ED) clinicians across the country are increasingly incorporating epinephrine, administered as an IV bolus, into their resuscitation practices. The technique, popularly referred to as push‐dose epinephrine (PDE), is extrapolated from air medical services and anesthesia practices.^1^, ^2^, ^3^ When indicated, it is broadly purposed to rapidly address hemodynamic instability in critically ill patients. However, the scarce literature and differing perspectives on PDE's safety have led to heterogenous implementation in practice.^4^

At our institution (a large urban academic hospital), the use of PDE in the ED has been intermittent and variable, with no supporting guidelines. Prompted by an internal medication‐use evaluation, our hospital's leadership called for a standardized process to optimize PDE's safe use. To describe the current landscape and inform development of a standardized process, we initiated a mixed methods project to survey and interview emergency medicine clinicians about the role of PDE in their practices.

We developed a six‐question, multiple‐choice survey asking about the availability and implementation of PDE at the respondent's practice site. In the fall of 2024, the electronic survey was shared across the Association of Academic Chairs of Emergency Medicine (AACEM) listserv (187 members). Each respondent was invited to also participate in an interview to discuss their perspective and experience. Snowball sampling was employed to recruit more clinicians. Survey and interview data underwent univariate and thematic analysis, respectively. Additional methodology is available in the Supplementary Methods.

In total, 23 emergency medicine clinicians completed the survey, including four by referral. The surveyed group represented practice from at least 13 different states. Since reporting of contact information was optional in the initial survey, we identify at least 19 department chairs or their delegated clinicians and at least 13 physicians and four pharmacists. Responses are featured in Table 1.

**TABLE 1:** Survey population.

Survey question^a^	Answer choice	*N*	(%)
Q1: Which option best describes your hospital's/institution's position on push‐dose epinephrine use in the emergency department? (*n* = 23)	Explicitly supported by policy, guideline, or protocol	9	(39)
	Explicitly prohibited	1	(4)
	No formal policies/procedures have been established	13	(57)
Q2: How is push‐dose epinephrine supplied at your practice site? (*n* = 23)	Prefilled syringes; 10 μg/mL	9	(39)
	Bedside preparation via dilution; 10 μg/mL	10	(43)
	Code syringe; 100 μg/mL	1	(4)
	Withdrawn from premixed bag; 32–64 μg/mL	1	(4)
	Not used	2	(9)
Q3: For which clinical indications does your practice site use push‐dose epinephrine? (*n* = 21; multiple responses)	Post–cardiac arrest hypotension	14	(67)
	Prevention of peri‐intubation hypotension	14	(67)
	Treatment of peri‐intubation hypotension	19	(90)
	Hypotension, as a bridge to continuous infusion	20	(95)
	Bradycardia	2	(10)
Q4: If push‐dose epinephrine is prepared on‐demand at the bedside, which option best describes the clinician(s) who prepare it? (*n* = 21)	Any clinician (physician, nurse, pharmacist)	4	(19)
	Physicians, typically or always	2	(10)
	Nurses, typically or always	1	(5)
	Pharmacists, typically or always	5	(24)
	No one. Dilutions are not performed bedside	9	(43)
Q5: Which option best describes your practice site's restrictions (either official or unofficial) on who may administer push‐dose epinephrine? (*n* = 21)	Only physicians may administer PDE	8	(38)
	Nurses, under physician supervision	8	(38)
	All clinicians, regardless of supervision	5	(24)
Q6: Does your practice site's emergency department utilize push‐dose PHENylephrine (“Neo”)? (*n* = 23)	Yes; it is supplied in prefilled syringes	18	(78)
	Yes, but it is prepared at the bedside	2	(9)
	No	3	(13)

^a^ Questions 1, 2, and 6 have 23 respondents. Questions 3, 4, and 5 have 21 respondents.

For the subsequent interview, 10 clinicians participated. Most were physicians, held leadership positions, and had been practicing for more than 20 years. Further details are available in Tables S1 and S2. Three primary themes pertaining to PDE emerged from the analysis. First, “implementation,” relates to the preparation, administration, dosing, and training with PDE. Second, “clinical value” focused on the clinician's perspective of literature and indications for PDE. Last, “errors & concerns” encompassed the clinician's perceived risks with PDE use.

*Implementation*. The majority of practice sites did not have PDE‐related guidelines or policies. When used, extemporaneous preparation of a 100 μg/10 mL syringe at the bedside was most common. At almost all practice sites, the pharmacist was at the center of PDE's preparation and handoff. Administration of PDE was usually tasked to nurses under physician supervision. The average dose was 10 to 20 μg (range 5 to 100 μg) given IV every 3 min (range 1 to 5 min). The median frequency of use was one dose per month, responses ranging from one to five.

There was general consensus that standardization of PDE was critical to its safe and effective use. One persisting threat is illustrated by ID‐23:

… it's hard at an academic medical center where you have rotators, off‐service residents, and different teams coming down to the emergency department … that adds an extra layer of difficulty … when the anesthesia residents are bringing syringes down in their pockets.

Clinical pharmacists were responsible for both bedside support and establishment of departmental safeguards. There was also evidence of reliance on pharmacists for operationalizing PDE, “…the only way that this is feasible is with the pharmacist” (ID‐11). Especially when pharmacists were not present, participants described the importance of supporting nursing services with clear instructions for preparation and labeling. For the physician residents, nine of the 10 participants described formal lectures as part of their training for PDE utilization, sometimes delivered by clinical pharmacists.

*Clinical value*. When asking participants how they first learned about PDE, four recalled training with “intravenous bolus epinephrine” for treatment of severe anaphylaxis (1980s–1990s). For two more recent graduates, PDE had been introduced as a general resuscitative tool. The remaining four learned about PDE informally.

When asked to characterize the literature on PDE use in the ED, responses ranged from “it doesn't exist” (ID‐24) to “it's widespread” (ID‐16). The most common responses were coded as equivocal and weak. One participant critiqued, “all of these studies characterized 50 or 100 mcg as a medication error… I don't think those are medication errors, because there are patients who need 50 mcg right now” (ID‐22).

Regarding PDE's role in the ED, most participants provided clinical indications, usually procedure‐related or as a temporizing measure. Additionally, two participants asserted PDE was essential to their practice, one elaborating: “it should be coming to the bedside a lot. It might not get pushed, but every time you have an intubation on patient's whose blood pressure is soft… procedural sedations in patients who are higher risk… we preemptively give PDE” (ID‐22). Conversely, two participants attested PDE having no role in their practice, citing “there's more risk than reward” (ID‐23) given the rapid availability of continuous infusions in well‐resourced ED's. Among participants who had phenylephrine available as prefilled syringes, PDE was preferential when desiring beta activity or treating anaphylaxis.

Some participants described urgency with PDE: “I need it now, not later” (ID‐16) and “I don't care if I overshoot. I cannot leave this patient at this blood pressure for one second longer” (ID‐22). Beyond that initial dose, one participant conveyed their summative view on PDE's clinical value:

I would say that anybody who utilizes push‐dose pressors for more than one administration is probably practicing bad medicine. Because if you can't get a mixed bag within 5 minutes to the bedside, that's poor practice. The assumption that one dose of push‐dose pressors is enough, is inappropriate, because you don't know. You might as well own a crystal ball. That is, to me, unimaginable that the practice of push‐dose pressors, which has been manifested in the anesthesia world for decades, has never been questioned or studied … we don't rapidly lower blood pressure because it harms patients. So why would we rapidly elevate blood pressure in a non‐titratable fashion? (ID‐24)

*Errors and concerns*. Most participants had not observed preparation errors, even though prefilled syringes were uncommon. Dosing and administration errors were observed by less than half of the participants. If errors were to occur, many participants expected adverse hemodynamic changes, while few believed harm was minimized by their established process. One participant suggested the possibility of cardiac arrest. With respect to the overall risk of errors occurring, there was a split between those concerned and those not concerned at all.

You're always going to have someone that's not familiar with it. We're [accustomed] to preparing drugs where the entirety gets pushed. And then you take a high‐stress scenario … (ID‐23)

Mixed properly, it has no negative clinical impact. You could give a full syringe of push‐dose epi, and you're never going to have a problem. Maybe they'll overshoot for a little while. (ID‐22)

The results of this quality improvement project illustrate the breadth of perspective and practice associated with PDE's use in the ED. A key finding from this investigation was that whether or not a departmental policy is adopted for PDE, there needs to be common understanding for its utilization across all disciplines. This includes education and training for all clinicians involved. Recognizing that PDE orders will likely be verbal, clear communication of the intended dose and instructive product labeling can help reduce errors. The preferred product is a prefilled syringe of epinephrine, 100 μg/10 mL, with usual dosing of 10 to 20 μg. Continued need for vasoactive support should be anticipated and come in the form of a continuous infusion. Whenever possible, system‐level structures to support safe and effective use are encouraged.

This study has limitations, most notably the small sample. However, the participants' decades of experience and geographical diversity are reassuring. Generalizability of the findings may be limited as the entire group was derived from academic medical centers, yet their viewpoints were most applicable to the goals of our project.

To our knowledge, there are no published reports describing survey or interview results regarding PDE utilization in the ED. There is a recent international survey and content analysis of pediatric critical care physicians using PDE in the intensive care unit setting.^5^ That group found PDE to be highly popular, although significantly heterogenous in application, with 100‐fold differences in dosing. They concluded research was needed to determine best practices, given the observed variability and unclear association with clinically important outcomes.

Though PDE is a popular resuscitation technique, results from our mixed methods nationwide survey illustrate heterogeneity in its application and differences in opinion on its role in therapy. Emergency medicine demands access to the tools deemed necessary for a patient's care, and optimal delivery of those tools begins with safety. The potential for PDE‐related harm is real^6^, ^7^, ^8^ but may be reduced by having a standardized, multidisciplinary process with prefilled syringes and quick access to continuous infusions. Ultimately, to best elucidate the risks and benefits of PDE in the ED, prospective randomized controlled trials would be needed.

## AUTHOR CONTRIBUTIONS

Andrew K. Posen, Shaveta Khosla, Terry Vanden Hoek, and Renee M. Petzel Gimbar each contributed to the study concept and design. Andrew K. Posen and Terry Vanden Hoek participated in the acquisition of data. Andrew K. Posen, Shaveta Khosla, Terry Vanden Hoek, and Renee M. Petzel Gimbar each contributed to the analysis and interpretation of data. Andrew K. Posen, Shaveta Khosla, and Renee M. Petzel Gimbar contributed to the drafting of the manuscript. Andrew K. Posen, Shaveta Khosla, Terry Vanden Hoek, and Renee M. Petzel Gimbar each partook in the critically important elements of manuscript revision. Shaveta Khosla served as the group's statistical expertise.

## CONFLICT OF INTEREST STATEMENT

The authors declare no conflicts of interest.

## Supporting information


Data S1.

## Data Availability

The data that support the findings of this study are available from the corresponding author upon reasonable request.
