# Supplementary material for: Current use of push‐dose epinephrine: Survey and interview results from academic clinicians in emergency medicine
Source: Acad Emerg Med. 2025 Mar 18;32(9):1017–20. doi: 10.1111/acem.70014 (PMC12435159; doi:10.1111/acem.70014)
Supplement: Supplementary file 1 — Data S1. [file ACEM-32-1017-s001.pdf]

SUPPLEMENTARY MATERIALS

Supplemental Methods

As described in the main text, this was a mixed methods project employing a survey and interview. Prospective interviewees were informed that participation was voluntary, recorded for transcribing purposes, and that their responses would be de-identified when presented. Interviews were conducted virtually, and followed a similar format, with minor modifications according to initial survey responses. Data were subjected to univariate and thematic analysis for the survey and interview, respectively. In the thematic analysis of open-ended questions, codes were generated through agreement of two independent coders with disagreement resolved by a third. Themes that emerged from the analysis were identified and an overview of themes was created. Additionally, qualitative content analysis was used on the transcribed data where open-ended questions were independently reviewed and grouped by the coders into categories and quantified. This study was conducted as a Quality Improvement project and received a 'not human subjects research' determination from the Institutional Review Board (STUDY2024-0970) at the [HOSPITAL].

Table S1: Interview Population and Practice Site Characteristics

Variable	Interview Results	N	%
Credential	Physician	7	70%
	Pharmacist	3	30%
Clinician Role	Leadership*	7	70%
	Non-leadership	3	30%
Years Post-Residency	1 to 9	2	20%
	10 to 19	3	30%
	20 or more	5	50%
ED Beds (include hall)	50 or less	2	20%
	51 to 99	4	40%
	100 or more	4	40%
Dedicated EM Pharmacist (multiple responses)	On Staff	10	100%
	7-day 24-hour coverage	2	20%
	Weekday, >50% hourly coverage	6	60%
	Full week, >50% hourly coverage	5	50%
Vasopressors stocked in ADS	Yes, at least 1 unique vasopressor	10	100%
	Yes, 2 or more unique vasopressors	10	100%
	Phenylephrine pre-filled syringes	7	70%

ADS=Automated Dispensing Cabinet

*All leadership roles held by these clinicians were either department heads or department chairs.

Table S2: Interview Results on Push-Dose Epinephrine Implementation

Variable	Interview Results	N	%
Push-Dose Epinephrine Product	Bedside preparation; 100 mcg/10 mL	7	70%
	Pre-filled syringe; 100 mcg/10 mL	3	30%
Role of PDE (multiple responses)	RSI; Adjunctive Support	5	50%
	Bridge to infusion	4	40%
	Transient hypotension-bradycardia*	3	30%
	Anaphylaxis	2	20%
	It is a necessity	2	20%
	No Role	2	20%
First learned about PDE	Bolus-Era Practice; Anaphylaxis	4	40%
	Residency	2	20%
	Discussion/Observation	3	30%
	Free Open-Access Medical education	1	10%
PDE Literature Characterization	Equivocal	3	30%
	Weak/Scarce	3	30%
	Sufficient/Supportive	2	20%
	Unfamiliar	1	10%
	Non-existent	1	10%
Resident PDE Training (multiple responses)	Formal Lecture	9	90%
	Informal Teaching	3	30%
	Live Observations	2	20%
	Simulation	1	10%
Relevance of PDE Dosing Errors (multiple responses)	Adverse Hemodynamic Changes	7	70%
	No Harm	3	30%
	Harm is minimized by established process	2	20%
	Risk of Cardiac Arrest	1	10%
	Patient-reported symptoms	1	10%
Concerns with PDE	Errors with preparation	4	40%
	Errors with dosing	2	20%
	No concerns	3	30%
	Patient harm	1	10%

PDE=Push-Dose Epinephrine; RSI=Rapid Sequence Intubation

*With no expectation to start an infusion
